# Differential Effect of Vaginal Microbiota on Spontaneous Preterm Birth among Chinese Pregnant Women

**DOI:** 10.1155/2022/3536108

**Published:** 2022-12-01

**Authors:** Hui Kan, Yining He, Qing Li, Yutong Mu, Yao Dong, Wei Fan, Miao Zhang, Tianlei Wang, Yaxin Li, Haiyan Liu, Anqun Hu, Yingjie Zheng

**Affiliations:** ^1^Department of Epidemiology, School of Public Health, Fudan University, Shanghai 200032, China; ^2^Key Laboratory for Health Technology Assessment, National Commission of Health and Family Planning, Fudan University, Shanghai 200032, China; ^3^Biostatistics Office, Clinical Research Unit, Shanghai Ninth People's Hospital, Shanghai Jiaotong University School of Medicine, Shanghai 200011, China; ^4^Department of Obstetrics and Gynecology, Anqing Municipal Hospital, Anqing 246003, China; ^5^Department of Clinical Laboratory, Anqing Municipal Hospital, Anqing 246003, China; ^6^Department of Blood Transfusion, Anqing Municipal Hospital, Anqing 246003, China; ^7^Laboratory of Public Health Safety, Ministry of Education, School of Public Health, Fudan University, Shanghai 200032, China

## Abstract

**Objective:**

The effect of vaginal microbiota on spontaneous preterm birth (sPTB) has not been fully addressed, and few studies have explored the associations between vaginal taxa and sPTB in the gestational diabetes mellitus (GDM) and non-GDM groups, respectively. *Study Design*. To minimize external interference, a total of 41 pregnant women with sPTB and 308 controls (pregnant women without sPTB) from same regain were enrolled in this case-cohort study. Controls were randomly selected at baseline. With the exception of GDM, other characteristics were not significantly different between the two groups. Vaginal swabs were collected at early second trimester. Using 16S amplicon sequencing, the main bioinformatics analysis was performed on the platform of QIIME 2. Vaginal microbiota traits of the sPTB group were compared with controls. Finally, the effects of binary taxa on sPTB in the GDM group and the non-GDM group were analyzed, respectively.

**Results:**

The proportion of GDM in the sPTB (19.51%) was higher than the controls (7.47%, *P* = 0.018). The vaginal microbiota of pregnant women with sPTB exhibited higher alpha diversity metrics (observed features, *P* = 0.001; Faith's phylogenetic diversity, *P* = 0.013) and different beta diversity metrics (unweighted UniFrac, *P* = 0.006; Jaccard's distance, *P* = 0.004), compared with controls. The presence of *Lactobacillus paragasseri/gasseri* (aOR: 3.12, 95% CI: 1.24-7.84), *Streptococcus* (aOR: 3.58, 95% CI: 1.68-7.65), or *Proteobacteria* (aOR: 3.39, 95% CI: 1.55-7.39) was associated with an increased risk of sPTB in the non-GDM group (*P* < 0.05). However, the relative abundance of novel *L. mulieris* (a new species of the *L. delbrueckii* group) was associated with a decreased risk of sPTB (false discovery rate, 0.10) in all pregnant women.

**Conclusion:**

GDM may modify the association of vaginal taxa with sPTB, suggesting that maternal GDM should be considered when using vaginal taxa to identify pregnant women at high risk of sPTB.

## 1. Introduction

Preterm birth (PTB) is considered a leading cause of neonatal mortality and morbidity worldwide [1, 2]. PTB is defined as a delivery before 37 gestation weeks. The majority of PTB cases occur in women without a clear risk factor and are classified as spontaneous PTB (sPTB) [2]. Unlike iatrogenic PTB (iPTB), sPTB is difficult to predict due to the lack of medical indicators [3].

It is believed that vaginal microbiota homeostasis plays an important role in maternal and infant health [4, 5]. Vaginal lactobacilli (*Lactobacillus jensenii*, *L. gasseri*, and *L. acidophilus*) show multimicrobial probiotic effect against dysbacteriosis. Although *L. jensenii* shows an individual probiotic effect against aerobic vaginitis, the protective effect of *L. gasseri* against bacterial vaginosis is often overlapped due to the copresence of other *Lactobacillus* species (*L. iners* and *L. crispatus*) [6]. Vaginal dysbacteriosis and some pathogens, such as *Chlamydia trachomatis*, *Ureaplasma* spp., or group B *Streptococcus* [5, 7], can cause adverse outcomes, including sPTB [8, 9]. Additionally, a meta-study showed that pregnant women with gestational diabetes mellitus (GDM) had a higher risk of sPTB than pregnant women without GDM [10]. Vaginal microbiota may play a complicated role in sPTB when GDM is considered.

Since 2011, vaginal microbiota has been classified into five community state types (CST) due to the dominant species, including CST-I (dominated by *L*. *crispatus*), II (*L. gasseri*), III (*L. iners*), and V (*L. jensenii*). Meanwhile, non*-Lactobacillus-*dominance type has been identified as CST-IV, a mixture of diverse taxa, e.g., *Atopobium*, *Prevotella*, and *Gardnerella* [11]. Due to the identification and isolation of *L. mulieris* from its closest relative *L. jensenii*, the CST-V dominated by *L. jensenii* might not be defined [12–14].

Vaginal microbiota exhibits race-specific characteristics, extending this effect further to sPTB [15]. The dominance species of CST-I, *L. crispatus*, tended to protect pregnant women from sPTB in a race-independent way [16–18]. However, the role of other specific taxa on sPTB was found to be inconclusive and varies in pregnant women of different races. It was further complicated by the fact that several studies did not yet differentiate sPTB from iPTB [19–21]. The risk of sPTB was found to decrease with *L. gasseri* or *L. jensenii* at the early second trimester in Caucasian-dominant women [22, 23] and at the third trimester in Indians [24]; but an increased risk with these two species at the late second-third trimester was also reported in the Caucasian-dominant women [25]. The risk increased with *L. iners* or CST-III in the second trimester or earlier in either African Americans [26, 27] or Caucasian-dominant women [28, 29]. It also increased with CST-IV at the first-second trimester in African Americans [26] and the second trimester in Koreans or Caucasian-dominant women [22, 23, 30, 31]. In addition, some vaginal non*-Lactobacillus* spp., including *Bacteroides*, *Prevotella*, BVAB1, *Sneathia amnii*, and *Atopobium*, have been reported to be associated with an increased risk of sPTB in varied racial pregnant women [18, 32, 33]. However, few studies have solicited the effect of significant vaginal taxa on sPTB in the GDM and non-GDM groups.

This study is aimed at investigating the differences in vaginal microbiota of pregnancies culminating in preterm versus term birth among Chinese pregnant women and at exploring the effects of vaginal taxa on sPTB in the GDM and non-GDM groups.

## 2. Materials and Methods

### 2.1. Study Design and Participants

We used a case-cohort design, whose source cohort had been described previously [14]. Briefly, pregnant women were recruited at their first prenatal visit to Anqing Municipal Hospital, Anhui Province, China, since February 22, 2018. Pregnant women (i) with informed consent, (ii) aged 18 years or older, (iii) in the first or early second trimester, (iv) not taking any antibiotics in the previous four weeks, and (v) absence of serious organic or systemic diseases (such as coronary heart disease, stroke, type 2 diabetes, and leukemia) were included. At enrollment, two vaginal samples were taken by skilled obstetricians, and a baseline questionnaire was used to collect patients' demographic, lifestyle, and clinical data. During follow-ups, any changes in the data since the last visit and complications from medical records were collected until delivery. Up to January 22, 2020, 1561 pregnant women (178 censors) were enrolled. GDM and sPTB were diagnosed by specialized doctors according to the results of the oral 75 g glucose tolerance test and gestation weeks, respectively [2, 34].

For this study, the controls were randomly selected at baseline using a systematic sampling method with a ratio of 1 : 4 (*n* = 390), and 308 with outcome out of the 390 pregnant women with qualified vaginal microbiota data were included in the final control group (Figure S1). Sixty-six pregnant women with PTB were obtained, of which 25 were excluded due to (i) iPTB (*n* = 15), (ii) unavailable microbiota data (*n* = 5), and (iii) multiple pregnancies (*n* = 5). Most characteristics were not significantly different between the two groups. A total of 349 pregnant women were available for analysis, including 41 singleton pregnant women with sPTB and 308 controls.

### 2.2. 16S rRNA Amplification and Sequencing

For vaginal samples in our study, the hypervariable V3-V4 region of the 16S rRNA gene was amplified and then sequenced on an Ion S5™ XL instrument (Novogene Co., Ltd., Beijing, China). Upstream analyses were performed on the platform of QIIME 2. Sequences were denoised by the DADA2 algorithm, and the representative sequences of amplicon sequence variants (ASVs) were annotated by the SILVA (version 138) database with an appropriate identity threshold (99%) [14, 35].

Sequences that lacked a good resolution at the species level were queried through the NCBI database with the local BLAST+ command [14, 17]; items with *E* values of <1 × 10^−50^, percentage of identical matches of >95%, and the highest bit score among each sequence were considered as the best matches [14]. Species items with more than one matching value were left blank, except for *L. paragasseri/gasseri*, which were highly identical sister taxa of the vaginal bacterial community [36]. Finally, the rarefied features according to the lowest number of reads (*n* = 3128) among the vaginal samples were retained. The upstream of MetaCyc pathway analyses was performed by the pipeline of PICRUSt2 (https://github.com/picrust/picrust2) [37].

### 2.3. Identification of Candidate Vaginal Bacterial Traits (VBTs)

We followed the steps to identify candidate VBTs. First, ASVs were collapsed to the same level. The discriminative taxa between sPTB and control groups were performed on the most abundant genus or species level, which met the following two criteria: (i) ≥5% of reads for at least one individual and (ii) ≥15% of pregnant women with nonzero data [38]. To reduce the redundancy of phenotypic information, higher-level taxa that had a high correlation (Spearman *r* > 0.985) estimated using the SparCC algorithm in FastSpar 0.0.10 with their corresponding lower-level taxa were excluded [39].

Second, 56 taxa (4 phyla, 6 classes, 7 orders, 9 families, 12 genera, and 18 species) were generated from the above processing. Specifically, the taxon with zero counts in more than 5% of the study samples was transformed to the presence/absence (P/A) trait, and those taxa that had a dominant threshold level (90%) of relative abundance (RAB) in more than 5% of the study samples were transformed to the dominance/none-dominance (D/ND) trait [14]. In addition to their transformed traits, the RAB of 56 taxa was naturally under consideration.

The alpha diversity metrics, including (i) ASV richness, (ii) the Shannon index, (iii) Faith's phylogenetic diversity, and (iv) Pielou's evenness, and beta diversity metrics including (i) the Bray-Curtis dissimilarity, (ii) weighted UniFrac, (iii) unweighted UniFrac, and (iv) the Jaccard distance were detected using the QIIME 2 diversity plugin. The CSTs of vaginal microbiota were clustered based on a Bray-Curtis distance matrix by Partitioning Around Medoids algorithm [40]. The 175 MetaCyc pathways, which had (i) no less than 5% of inferred abundance for at least one individual and (ii) no less than 15% of pregnant women without zero data, were retained.

### 2.4. Statistical Analysis

Differences of alpha and beta diversity metrics of vaginal microbiota between the sPTB and control groups were estimated using the Wilcoxon test and principal coordinates analysis (PCoA), respectively. The differences of RAB of MetaCyc pathways between the sPTB and controls were evaluated using Welch's *t*-test in STAMP [41].

According to the literature review [42–44] and the actual data-generating process in our study, five variables (age, number of previous pregnancies, prepregnancy body mass index (BMI), passive smoking, and GDM) were included as adjustment variables. Further analyses and visualizations were performed using R 4.0.3. The missing values of passive smoking (15%) and prepregnancy BMI (1%) were imputed with the mice package based on a random forest model [14, 45]. The differences of RAB of taxa between the sPTB and control groups were compared using a zero-inflated negative binomial linear effect model (in the pscl package) due to the distribution features [46, 47], and the adjusted coefficients (aCoef) and 95% confidence interval (CI) were retained. The binary logistic regression model was performed for screening CSTs and binary taxa, and the adjusted odds ratio (aOR) and 95% CI were retained [48]. The level of a statistical significance was set at a two-sided *P* value of 0.05, and the false discovery rate (FDR) was set at 0.20. The visualization of correlations between taxa and MetaCyc pathways was evaluated using the Spearman test in the corrplot package [49]. Additionally, the Kaplan-Meier survival analysis (in the survival and survminer packages) and logistic model (in the stats package) were performed to explore the associations between binary taxa and sPTB in the GDM and non-GDM groups [32, 50].

## 3. Results

### 3.1. Description of the Participants

From the first trimester until delivery, data available for our final analysis (*n* = 349) included 41 pregnant women in the sPTB group and 308 in the control group. The majority of them (98%) at baseline were absent of vaginitis according to white blood cell counts and *Trichomonas* and yeast tests. With the exception of GDM, other characteristics (age, gestational age at baseline, prepregnancy BMI, ethnicity, education level, periodontitis, vaginal douche habit, and passive smoking) were not significantly different between the two groups. The proportion of GDM in the sPTB (19.51%) was higher than the controls (7.47%, Table 1).

### 3.2. Diversity and CSTs

**Figure 1:**
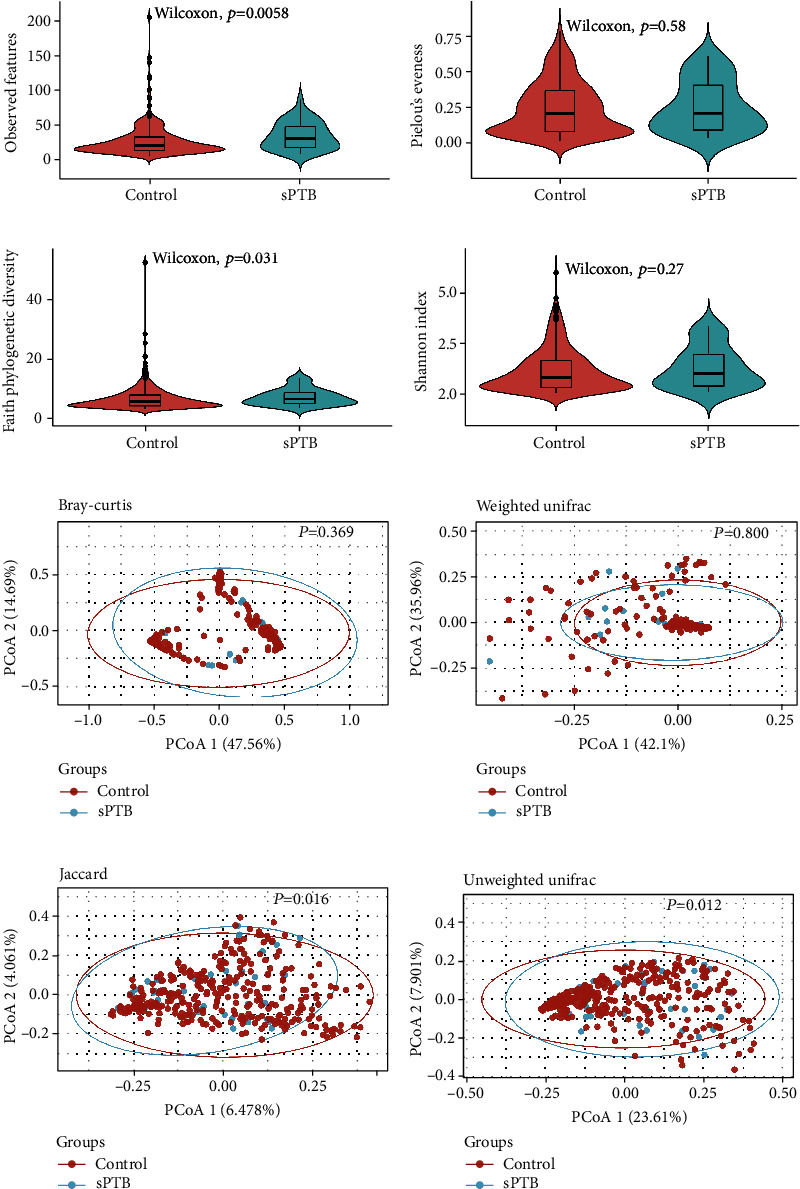
The alpha and beta diversity metrics of vaginal microbiota between the sPTB and controls. The violin plots were applied to illustrate the alpha diversity metrics (a–d) between the two groups. *P* values in the violin plots were evaluated based on the Wilcoxon test. PCoA plots were applied to illustrate the beta diversity metrics (e–h) between the two groups, and *P* values in the plots were evaluated based on the Adonis analysis. The percentage on axis label was the proportion of variance explained by that axis.

The vaginal microbiota of pregnant women with sPTB exhibited higher observed features (*P* = 0.006) and Faith's phylogenetic diversity (*P* = 0.031), compared with controls. The beta diversity metrics of unweighted UniFrac and Jaccard's distance were different between the sPTB and controls (Figure 1).

The vaginal CSTs were clustered into four groups, CST-I (dominated by *L. crispatus*), CST-II (*L. paragasseri/gasseri*), CST-III including two subtypes: CST-IIIa (*L. iners*) and CST-IIIb (a mixture of dominant *L. iners* with *Gardnerella* spp., *L. paragasseri/gasseri*, and other microbiotas), and CST-IV without any dominant microbiota but a mixture of diverse species, including *Gardnerella* spp. and *Fannyhessea vaginae* (formerly known as *Atopobium vaginae*) (Figure S2). The highest proportion of CSTs among all pregnant women was CST-I (35.53%), followed by CST-IIIa (28.94%), CST-IV (17.48%), CST-IIIb (13.75%), and CST-II (4.30%).

### 3.3. Associations of VBTs with sPTB

A total of 56 taxa passed the prescreening and were included in the subsequent analyses. Three binary taxa, including *Streptococcus* (P/A) (aOR: 2.98, 95% CI: 1.49-5.97, *P* = 0.002, *P*_FDR_ = 0.069), *Streptococcaceae* (P/A) (aOR: 2.92, 95% CI: 1.46-5.85, *P* = 0.002, *P*_FDR_ = 0.069), and *Proteobacteria* (P/A) (aOR: 2.48, 95% CI: 1.24-4.97, *P* = 0.010, *P*_FDR_ = 0.194), were associated with an increased risk of sPTB (*P*_FDR_ < 0.20). No significant CST was linked to sPTB compared to CST-I.

**Figure 2:**
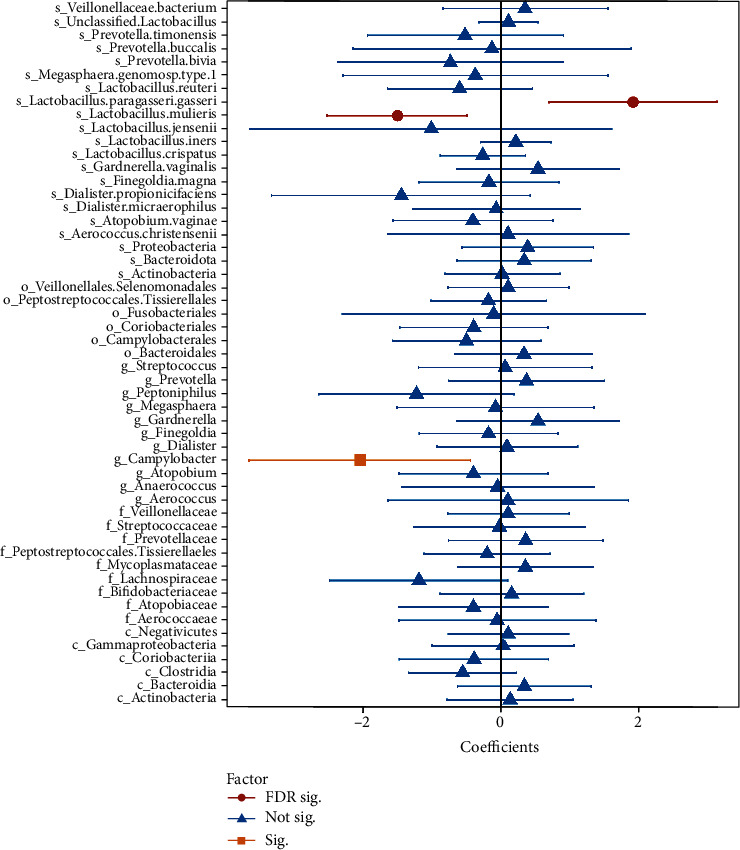
Forest plots of vaginal bacterial traits associated with sPTB. The difference of the relative abundance of vaginal taxa between the sPTB and controls was evaluated using a zero-inflated negative binomial linear effect model. Shapes (red circles, FDR < 0.20; orange squares, raw *P* < 0.05; blue triangles, raw *P* ≥ 0.05) represent estimated coefficients, and lines show the 95% CIs of the coefficients.

**Figure 3:**
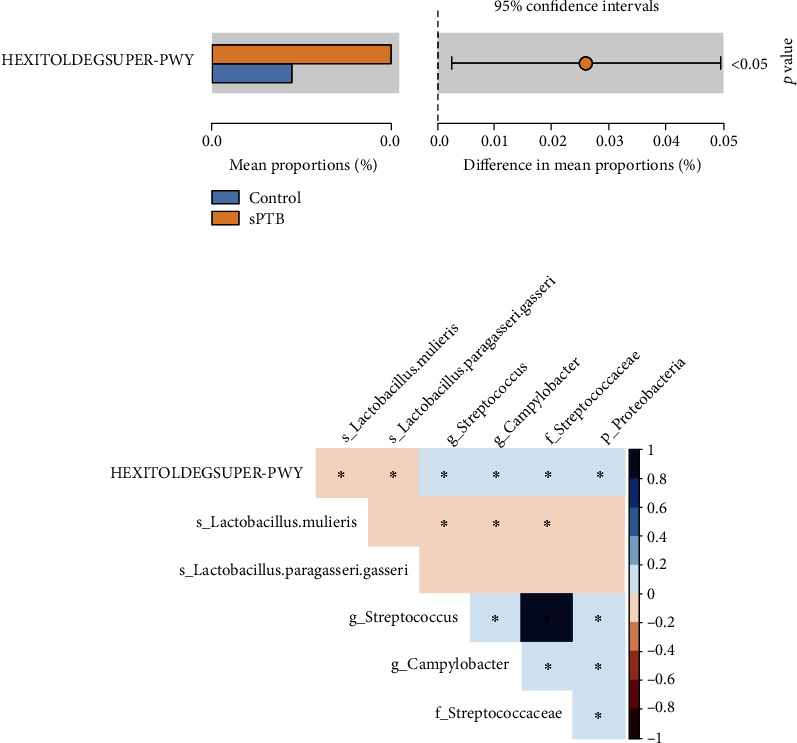
Correlation between superpathway of hexitol degradation and significant taxa. (a) The difference of superpathway abundance of hexitol degradation between the sPTB and controls was evaluated using Welch's *t*-test (*P* < 0.05). (b) The relative abundance of taxa linked to sPTB was analyzed for covariation with superpathway of hexitol degradation using the Spearman test. Correlation effects were color-coded, from red (negative correlation) to blue (positive correlation). ^∗^*P* < 0.05.

The RAB of novel *L. mulieris* (aCoef = −1.50; *P* = 0.004) was negatively associated with sPTB, but the RAB of *L. paragasseri/gasseri* (aCoef = 1.90, *P* = 0.002) was positively associated with sPTB (Figure 2). The RAB of HEXITOLDEGSUPER-PWY pathway (superpathway of hexitol degradation) was different between the sPTB and controls (*P* < 0.05, Figure 3(a)), and the RAB of *Proteobacteria*, *Streptococcaceae*, *Streptococcus*, *L. mulieris*, and *L. paragasseri/gasseri* was correlated to the superpathway abundance of hexitol degradation (*P* < 0.05, Figure 3(b)).

### 3.4. Cumulative Hazard of sPTB among Pregnant Women in Different Strata

**Figure 4:**
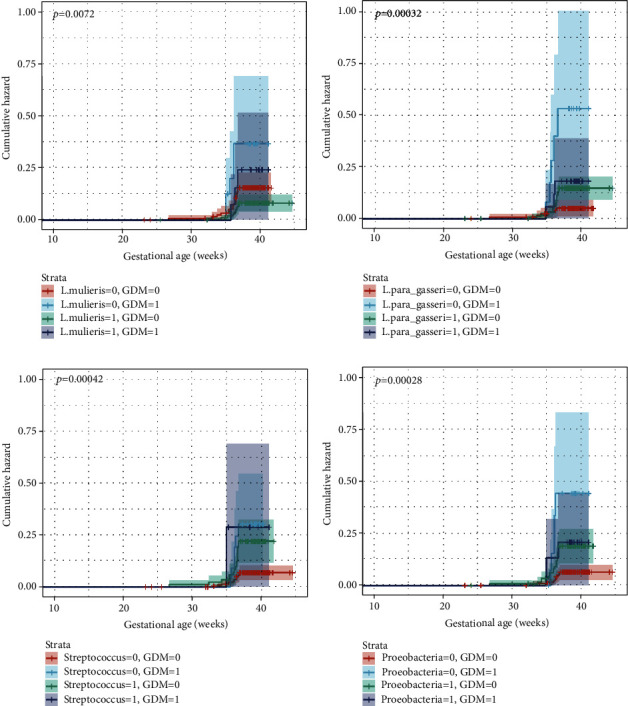
Cumulative hazard of sPTB among pregnant women in different strata. The taxa and GDM were treated as binary traits where 0 and 1 represented the absence and presence of vaginal taxa or GDM, respectively. *P* values were retained from log-rank analyses. (a) *L. mulieris*. (b) *L. paragasseri/gasseri*. (c) *Streptococcus*. (d) *Proteobacteria*.

The survival curve results implied the interactions of vaginal taxa (*L. mulieris*, *L. paragasseri/gasseri*, *Streptococcus*, and *Proteobacteria*) and GDM on sPTB (*P* < 0.05, Figure 4). The aOR of interaction term between *L. paragasseri/gasseri* and GDM on multiplicative scale was 0.10 (95% CI: 0.01-0.68, *P* = 0.019), and the aOR of interaction term between *Proteobacteria* and GDM was 0.15 (95% CI: 0.02-0.99, *P* = 0.048, Table S1). The presence of vaginal *L. paragasseri/gasseri* (aOR: 3.12, 95% CI: 1.24-7.84), *Streptococcus* (aOR: 3.58, 95% CI: 1.68-7.65), and *Proteobacteria* (aOR: 3.39, 95% CI: 1.55-7.39) increased the risk of sPTB in the non-GDM group but not in the GDM group (Table 2).

## 4. Discussion

Our study presented a complete picture of the vaginal microbiota associated with sPTB, aided by refined classification schemes for both VBTs and PTB. The vaginal microbiota of pregnant women with sPTB exhibited different diversity metrics and hexitol degradation abundance compared to controls. Vaginal *L. mulieris* decreased the risk of sPTB in all pregnant women. Vaginal *L. paragasseri/gasseri*, *Streptococcus*, and *Proteobacteria* increased the risk of sPTB in the non-GDM group.

The differences in diversity metrics of vaginal microbiota between the sPTB and controls indicated their different vaginal microbiota compositions in our study, which were supported by other infection diseases [51, 52]. It is acknowledged that vaginal microbiota dominated by *Lactobacillus* spp. is “good” for pregnancy, but it is not that simple [15]. A recent study evidences a probiotic multimicrobial consortium by *Lactobacillus* species (*L. iners*, *L. jensenii*, *L. gasseri*, and *L. acidophilus*) against vaginal dysbiosis. In addition, the presence of *L. acidophilus* and *L. gasseri* in other *lactobacillus* clusters may enhance probiotic protection in vaginal dysbiosis establishment. Vaginal *L. jensenii* shows a probiotic effect on aerobic vaginitis individually, but the protective effect of *L. gasseri* against bacterial vaginosis is overlapped due to the copresence of other *Lactobacillus* species, especially *L. iners* and *L. crispatus* [6].

First, our study identified a depleted RAB of *L. mulieris*, a newly discovered and *L. jensenii* genetically related species in 2020 [13, 14], in sPTB cases. A decreased risk of sPTB with *L. jensenii* in Australians but an increased risk has been reported in Caucasians [23, 25, 30]. In our study, we were unable to duplicate the findings of the associations mentioned above. It implied that the real species linked to sPTB might be *L. mulieris* but not *L. jensenii* in Chinese pregnant women. Some studies showed that *L. mulieris* was a member of *Lactobacillus* spp., like *L. crispatus*, which produces some antimicrobial substances (such as hydrogen peroxide, antimicrobial peptides, and biosurfactants) and promotes local immunity to reduce adhesion and colonization of pathogenic microorganisms [12, 53–55]. Similar to *L. crispatus*, the antibacterial ability of *L. mulieris* ensured the low diversity of vaginal microbiota, which tended to decrease the risk of sPTB. The protective role of *L. mulieris* on sPTB was detected in our study, which was rarely noticed.

Second, *L. gasseri* has a sister strain *L. paragasseri*, and a large portion of genomes labelled as *L. gasseri* currently should be reclassified as *L. paragasseri* [36, 56]. In this study, *L. paragasseri/gasseri*, classified as one feature according to the 16S rRNA gene [14], increased the risk of sPTB as supported by one study undertaken in the late second trimester [25]. However, other studies undertaken in the first or early second trimester tended to report a protective role of *L. gasseri* on sPTB among Caucasian-dominant women [43]. Generally, the presence of *L. gasseri* was likely to fluctuate over time, be positively correlated with higher concentrations of proinflammatory cytokines in the vaginal fluid [57], and predispose to some extent to the abnormal vaginal microbiota [58].

Third, *Streptococcus* and *Proteobacteria* were also found to be associated with an increased risk of sPTB. Group B *Streptococcus*, one of the species within the *Streptococcus*, is a commensal bacterium of the vagina [59]. It can be transformed from an asymptomatic carriage state into a bacterial pathogen for adverse pregnancy outcomes [60], including PTB [8]. The presence of *Proteobacteria* was reported to reflect the dysbiosis or an unstable microbial community structure, tending to become colitogenic microbes that can trigger inflammatory responses [61, 62]. A sow study showed that vaginal *Proteobacteria* was more abundant in sows with endometritis than those healthy sows [62].

Furthermore, our study suggested that the abundance of HEXITOLDEGSUPER-PWY pathway (superpathway of hexitol degradation) in vaginal microbiota was associated with sPTB. Bacteria can utilize hexitols as a source of carbon and energy; hence, a high abundance superpathway of hexitol degradation reflects a high load of bacteria [63]. A recent study showed that the high load bacteria in vagina might trigger the sPTB. When vaginal bacteria are densely concentrated, they will travel through the cervical mucus plug and enter the upper genital tract, which may cause inflammation [64]. Additionally, the superpathway of hexitol degradation in the gut microbiota tends to decrease among Crohn's disease patients achieving remission, suggesting the hexitol degradation is associated with immune responses [65, 66]. Hexitol production and accumulation has been implicated in the pathogenesis of diabetic complications [67]. Hexitol production can lead to the basement membrane thickening in microvessels, which is demonstrated in diabetic microvascular disease [63, 67, 68]. The basement membrane thickening in fetal membranes will lead to an increase in membrane fragility and premature rupture of membranes. The high load bacteria, immune responses, and membrane fragility associated with hexitol degradation may participate in the etiology of sPTB. However, further research must be done to determine the precise factors causing sPTB. This study showed that vaginal *L. paragasseri/gasseri* and *L. mulieris* were negatively associated with the superpathway of hexitol degradation, and *Streptococcus* and *Proteobacteria* were positively associated with the superpathway of hexitol degradation. Vaginal *L. paragasseri/gasseri*, *Streptococcus*, and *Proteobacteria* increased the risk of sPTB in the non-GDM group but not in the GDM group (Table 2), suggesting the antagonism of vaginal taxa (*L. paragasseri/gasseri*, *Streptococcus*, and *Proteobacteria*) and GDM on sPTB. Vaginal microbiota and GDM are both linked to sPTB, implying their complex roles in these issues [69–71].

GDM is a state of chronic, low-grade inflammation [72], evidenced by the increased levels of tumor necrosis factor-*α* and interleukin-6 in pregnant women with GDM [73–76]. The inflammatory response was reported to be reduced by selenases (selenium containing enzymes), and low blood selenium levels in pregnant women decreased the risk of both GDM and sPTB [77, 78]. GDM may affect the vaginal microbiota of women during pregnancy through inflammation [34], which warrants further investigation.

Our studies have some implications. (i) The role of vaginal microbiota on sPTB should be integral or interactive. (ii) The combination of these taxa may help us identify pregnant women who are at high risk of sPTB. (iii) New questions were open for future studies, e.g., the exact species of *Streptococcus* or *Proteobacteria* linked to sPTB and their effects on sPTB in GDM stratification.

Our study has several strengths. (i) The prospective nature of our case-cohort design reduced information bias. (ii) Our analysis was limited to pregnant women with sPTB by excluding those with iPTB, making our sPTB group more clinically homogeneous. (iii) We presented a relatively complete picture of the association between VBTs and sPTB. Our study also has limitations. A detailed evaluation of vaginal swabs was not done, such as pH value. The association between vaginal microbiota and sPTB was investigated in singleton pregnant women. When this association is expanded to multiple pregnancies, further research needs to be done. Additionally, only one vaginal sample was collected of each pregnant woman, and this study has absence of longitudinal analysis between vaginal microbiota and sPTB.

## 5. Conclusions

Our comprehensive study showed that the diversity matrixes of vaginal microbiota were different between the sPTB and controls; the risk of sPTB decreased with vaginal *L. mulieris* in all pregnant women but increased with *L. paragasseri/gasseri*, *Streptococcus*, and *Proteobacteria* in the non-GDM group. The findings suggest that maternal GDM should be considered when using vaginal taxa to identify pregnant women at high risk of sPTB.

## Figures and Tables

**Table 1:** Characteristics of study participants between the sPTB and controls.

Characteristics	Controls	sPTB	*P* ^a^
Age (years)	28 (26-30)^b^	28 (25-30)	0.660
Gestational age at baseline (weeks)	16.43 (15.57-17.00)	16.29 (13.86-16.57)	0.153
Prepregnancy BMI	20.95 (19.31-22.77)	20.96 (19.53-23.63)	0.989
Ethnicity			
Chinese Han	305 (99.03)^c^	40 (97.56)	0.395
Education level			
Primary/junior high school	80 (25.97)	6 (14.64)	0.141
High school	56 (18.18)	12 (29.27)	
College or above	172 (55.84)	23 (56.10)	
Periodontitis			
No	220 (71.43)	32 (78.05)	0.085
Bleeding but untreated	68 (22.08)	4 (9.76)	
Diagnosed and treated	11 (3.57)	4 (9.76)	
Diagnosed but untreated	9 (2.92)	1 (2.44)	
Vaginal douche habit			
Never	253 (82.14)	30 (73.17)	0.209
Ever but no more	17 (5.52)	5 (12.20)	
Yes	38 (12.34)	6 (14.63)	
Passive smoking^d^			
<1 day/week	242 (78.57)	33 (80.49)	0.737
1-3 days/week	34 (11.04)	6 (14.63)	
3-6 days/week	4 (1.30)	0 (0.00)	
Nearly everyday	28 (9.09)	2 (4.88)	
GDM			
No	285 (92.53)	33 (80.49)	0.018

^a^ *P* values for continuous variables were calculated using the Wilcoxon test, and *P* values for categorical variables were calculated using the Fisher test. ^b^Median (interquartile range). ^c^Number (percentage). ^d^Passive smoking was measured by days per week with passive smoking over 15 minutes. BMI: body mass index (kg/m^2^); GDM: gestational diabetes mellitus.

**Table 2:** The associations between vaginal taxa and sPTB in the GDM and non-GDM groups.

Vaginal taxa (P/A)	Non-GDM (*n* = 318)				GDM (*n* = 31)			
	cOR (95% CI)	*P*	aOR (95% CI)^a^	*P*	cOR (95% CI)	*P*	aOR (95% CI)^a^	*P*
*p_Proteobacteria*	2.85 (1.36-5.96)	0.006	3.39 (1.55-7.39)	0.002	0.46 (0.09-2.41)	0.359	0.65 (0.09-4.48)	0.659
*g_Streptococcus*	3.76 (1.80-7.85)	0.0004	3.58 (1.68-7.65)	0.001	0.94 (0.15-6.01)	0.952	1.30 (0.16-10.45)	0.802
*s_L.para.gasseri*	2.66 (1.12-6.34)	0.027	3.12 (1.24-7.84)	0.016	0.32 (0.06-1.70)	0.181	0.30 (0.05-1.87)	0.197
*s_L.mulieris*	0.49 (0.24-1.02)	0.057	0.54 (0.26-1.13)	0.101	0.65 (0.13-3.40)	0.614	0.42 (0.07-2.61)	0.354

^a^The association between vaginal taxa and sPTB was estimated with logistic model adjusted for age, number of previous pregnancies, prepregnancy body mass index, and passive smoking. aOR: adjusted odds ratio; CI: confidence interval; cOR: crude odds ratio; GDM: gestational diabetes mellitus; P/A: presence/absence.

## Data Availability

The data presented in this study are available from the corresponding authors (zhengshmu@gmail.com) on reasonable request.

## Supplementary Materials

Supplementary Materials Figure S1: the flowchart for the process of pregnant women enrolled in this study. Figure S2: vaginal bacterial composition in the sPTB and controls. Table S1: interaction of GDM and vaginal taxa on sPTB.
