# Supplementary material for: Differential Effect of Vaginal Microbiota on Spontaneous Preterm Birth among Chinese Pregnant Women
Source: Biomed Res Int. 2022 Dec 1;2022:3536108. doi: 10.1155/2022/3536108 (PMC9731763; doi:10.1155/2022/3536108)
Supplement: Supplementary Materials — Figure S1: the flowchart for the process of pregnant women enrolled in this study. Figure S2: vaginal bacterial composition in the sPTB and controls. Table S1: interaction of GDM and vaginal taxa on sPTB. [file 3536108.f1.pdf]

Contents

Supplementary figures 2

Figure S1. The flowchart for the process of pregnant women enrolled in this study..2

Figure S2. Vaginal bacterial composition in the sPTB and controls................3

Supplementary tables 4

TableS1 Interaction of GDM and vaginal taxa on sPTB. 4

Supplementary figures

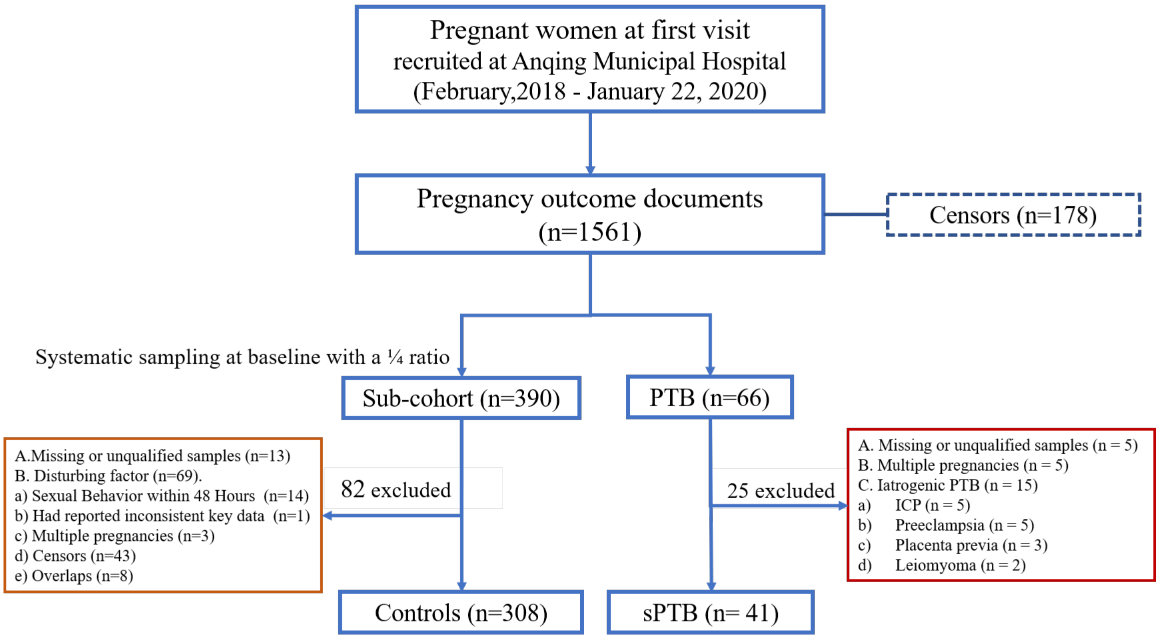

Figure S1. The flowchart for the process of pregnant women enrolled in this study. PTB, preterm birth; sPTB, spontaneous preterm birth; ICP, intrahepatic cholestasis of pregnancy.

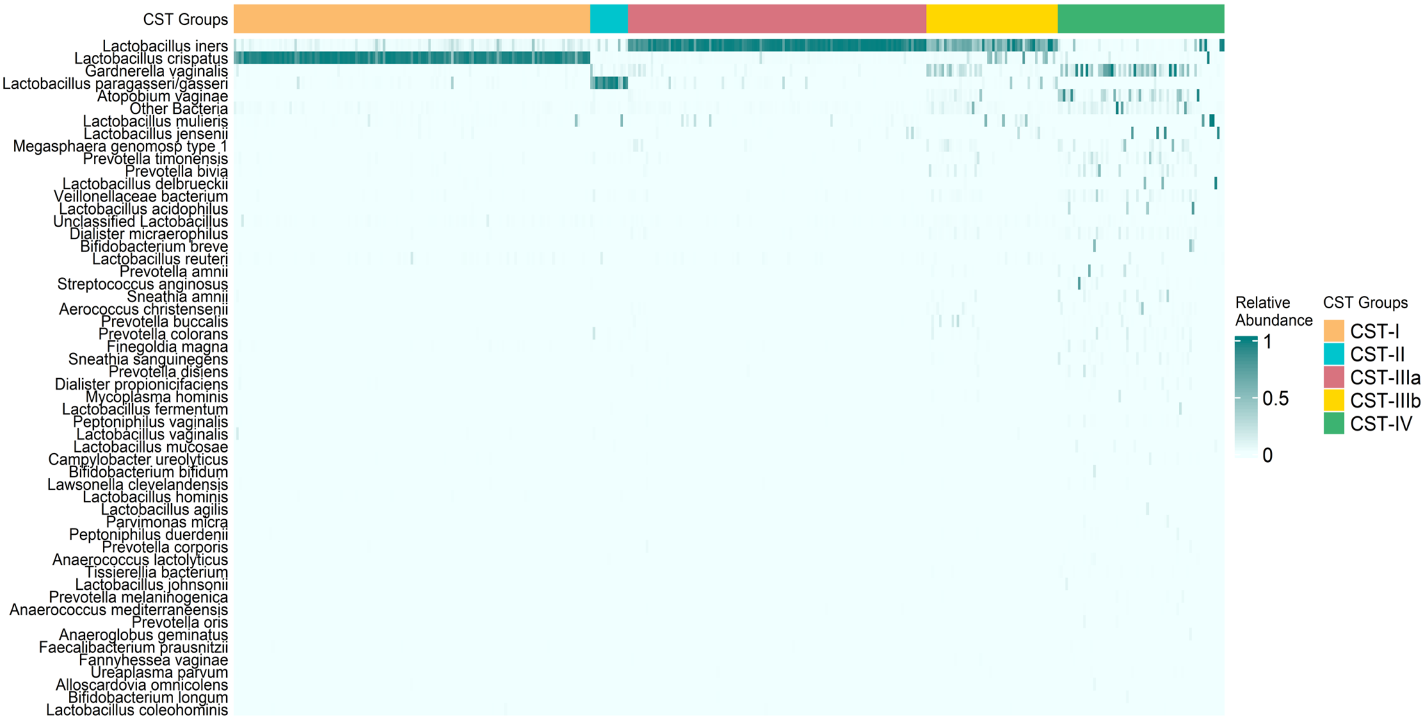

Figure S2. Vaginal bacterial composition in the sPTB and controls. The composition of vaginal microbiota among CSTs were exhibited at species level, and different CSTs annotated with distinction colors were marked by the dominant species. CSTs, community state types; sPTB, spontaneous preterm birth.

Supplementary tables

Table S1 Interaction of GDM and vaginal taxa on sPTB.

Terms	Multiplicative scale		
	aOR(95%CI)[a]	*P*	FDR[b]
g_Streptococcus:GDM[c]	0.29(0.04-2.19)	0.230	0.767
p_Proteobacteria:GDM	0.15(0.02-0.99)	**0.048**	0.161
s_L.mulieris:GDM	1.10(0.18-6.84)	0.920	0.990
s_L.paragasseri/gasseri:GDM	0.10(0.01-0.68)	**0.019**	0.062

[a]aOR, adjusted odds ratio of each interaction term in logistic model was adjusted for age, number of previous pregnancies, pre-pregnancy body mass index, passive smoking; CI, confidence interval. [b]FDR, False discovery rate. [c] GDM, gestational diabetes mellitus.
